# Development and Clinical Application of Positron Emission Tomography Imaging Agents for Monoamine Oxidase B

**DOI:** 10.3389/fnins.2021.773404

**Published:** 2022-02-25

**Authors:** Jeffrey H. Meyer, Joeffre Braga

**Affiliations:** ^1^Brain Health Imaging Centre, Centre for Addiction and Mental Health, Toronto, ON, Canada; ^2^Department of Psychiatry, University of Toronto, Toronto, ON, Canada; ^3^Department of Pharmacology and Toxicology, University of Toronto, Toronto, ON, Canada

**Keywords:** MAO-B, astrogliosis, PET imaging, monoamine oxidase, depression, smoking, [^11^C]L-deprenyl, [^11^C]SL25.1188

## Abstract

Monoamine oxidase B (MAO-B) is a high-density protein in the brain mainly found on outer mitochondrial membranes, primarily in astroglia, but additionally in serotonergic neurons and in the substantia nigra in the midbrain. It is an enzyme that participates in the oxidative metabolism of important monoamines including dopamine, norepinephrine, benzylamine, and phenylethylamine. Elevated MAO-B density may be associated with astrogliosis and inhibiting MAO-B may reduce astrogliosis. MAO-B density is elevated in postmortem sampling of pathology for many neuropsychiatric diseases including Alzheimer’s disease, Parkinson’s disease, Huntington’s disease, amyotrophic lateral sclerosis, and alcohol use disorder. Initial development of positron emission tomography (PET) imaging agents focused on analogs of [^11^C]L-deprenyl, with the most commonly applied being the deuterium substituted [^11^C]L-deprenyl-D2. This latter radiotracer was modeled with an irreversible trapping compartment reflecting its irreversible binding to MAO-B. Subsequently, [^11^C]SL25.1188, a reversible binding MAO-B radioligand with outstanding properties including high specific binding and excellent reversibility was developed. [^11^C]SL25.1188 PET was applied to discover a substantive elevation of MAO-B binding in the prefrontal cortex in major depressive disorder (MDD) with an effect size of more than 1.5. Longer duration of MDD was associated with greater MAO-B binding throughout most gray matter regions in the brain, suggesting progressive astrogliosis. Important applications of [^11^C]L-deprenyl-D2 PET are detecting a 40% loss in radiotracer accumulation in cigarette smokers, and substantial occupancy of novel therapeutics like EVT301 and sembragiline. Given the number of diseases with elevations of MAO-B density and astrogliosis, and the advance of [^11^C]SL25.1188, clinical applications of MAO-B imaging are still at an early stage.

## Introduction

This review starts with a background regarding the importance of monoamine oxidase B (MAO-B) function and role in neuropsychiatric illness; followed by development of PET imaging agents, focusing on those reaching human use. This is followed by discussion of radiotracers metabolized by MAO-B or irreversibly bound to MAO-B; vs. radioligands reversibly bound to MAO-B; and finally, some key clinical applications of MAO-B imaging. Background information will focus on the cellular and brain distribution of MAO-B, its functional role, abnormalities in postmortem tissue of neuropsychiatric diseases, and therapeutic inhibitors. Development of imaging agents will focus on [^11^C]L-deprenyl analogs that use a radiotracer trapping approach for quantitation and [^11^C]SL25.1188 which reversibly binds to MAO-B. Advantages and disadvantages of these approaches will be discussed. Clinical applications, mostly of quantitative methods, are then presented in major depressive disorder (MDD), cigarette smoking, and occupancy.

## Monoamine Oxidase B

Monoamine oxidase has two isoforms in mammals, monoamine oxidase A and MAO-B, encoded by separate genes with about 70% common identity. MAO-B is an enzyme of 520 amino acids and is found in most tissues in the body including the brain (Saura et al., 1992, 1996). The structure of the human MAO-B enzyme includes an entrance cavity of 290A and a substrate cavity of 400A. In humans, MAO-B is found in dimers (Binda et al., 2011). In the brain, the predominant location for this enzyme is on the outer mitochondrial membranes of astrocytes and, within the midbrain, serotoninergic neurons, although there is also some MAO-B in dopamine containing cells within the substantia nigra (Damier et al., 1996; Saura et al., 1996; Arango et al., 2002; Lopez-Munoz and Alamo, 2009; Rajkowska and Stockmeier, 2013). In human brain, MAO-B is a high density target, abundant across many brain regions ranging from 1 to 5 ng/μg protein (and is two- to threefold higher than MAO-A) (Tong et al., 2013). MAO-B density is highest in the raphe nuclei in the brainstem (Saura et al., 1992, 1996; Tong et al., 2013). Available evidence indicates that MAO-B expression within these serotonergic neurons is largely confined to the nuclei, possibly because mitochondria with MAO-B are not transported to serotonergic nerve terminals (Arai et al., 2002). The serotonergic neurons of the raphe are responsible for most of the efferent serotonergic projections to the brain. Additionally, MAO-B density is very high in striatum and thalamus, high in the cortex and hippocampus, and relatively lower in cerebellar cortex and white matter tissue (Saura et al., 1992, 1996; Tong et al., 2013). Given the relatively lower expression of MAO-B in white matter, that fibrous astrocytes are mainly in white matter and that protoplasmic astrocytes are mostly found in gray matter, it could be posited that MAO-B is expressed more in protoplasmic astrocytes. However, it is also noteworthy that MAO-B has been identified in both protoplasmic and fibrous astrocytes (Levitt et al., 1982; Saura et al., 1996).

**FIGURE 1:**
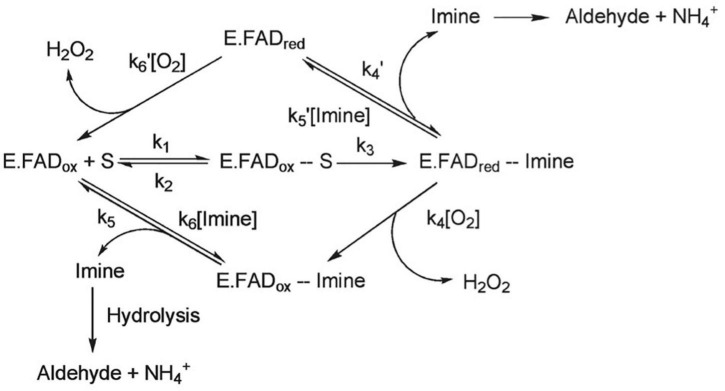
MAO-B can follow one of two general catalytic reactions, however, for most substrates, the pathway depicted on the bottom of the figure is followed. In this pathway, oxygen reacts with the enzyme-product complex. It is believed that the substrate binds to the active site on MAO-B via a deprotonated amine moiety, where it is oxidized to a protonated imine and the covalent FAD cofactor is reduced to its hydroquinone form. FAD cofactor then reacts with O_2_ to generate flavin and hydrogen peroxide, and an aldehyde is formed when the protonated imine is released from the enzyme and undergoes hydrolysis. Adapted with permission from Edmondson et al. (2009). Copyright © (2009) American Chemical Society.

MAO-B has an important role in oxidation of amines including dopamine, norepinephrine, benzylamine, and phenylethylamine. The main catalytic reaction pathway involves oxygen reacting with the enzyme-product complex (see Figure 1). At the end of the pathway reduction of cofactor flavin adenosine dinucleotide reacts with O_2_ to generate flavin and hydrogen peroxide (Binda et al., 2011). The metabolism of monoamines and production of hydrogen peroxide as well as aldehydes are of particular interest for understanding the relationship of MAO-B function to brain health. It is also proposed that MAO-B might facilitate production of toxic metabolic products as exemplified after MPTP administration. MPTP (1-methyl-4-phenyl-1,2,5,6-tetrahydropyridine) was originally a contaminant in the (illegal) synthesis of opiate desmethylprodine that induced Parkinson’s disease symptoms. Administration of MPTP in rodents results in behaviors of reduced and slowed movement which do not occur when MAO-B inhibitors are administered, implying that MAO-B metabolism of MPTP is required for the toxicity (Heikkila et al., 1984; Kupsch et al., 2001). However, it is noted that rats are not as susceptible to MPTP toxicity as mice (Lee et al., 1992). Generating oxidative stress adjacent to mitochondrial membranes and creation of potentially neurotoxic metabolic products has implicated a role for MAO-B in apoptosis and neurodegeneration (Riederer et al., 2004; Youdim et al., 2006; Mallajosyula et al., 2008).

The effects of overexpression of MAO-B and MAO-B knockout have also been evaluated. In a transgenic mouse model of globally increased MAO-B in astrocytes, behavioral changes during open field assessment included reduced total movement, less distance traveled, lower movement speed, and lower duration of time moving, effects attributed to midbrain neurodegeneration but it is noteworthy that the MAO-B overexpression was global across astrocytes throughout the brain (Mallajosyula et al., 2008; Siddiqui et al., 2011). The astrocytosis associated with MAO-B overexpression in this transgenic model was prevented by accompanying MAO-B inhibitor treatment (Mallajosyula et al., 2008; Siddiqui et al., 2011). In a knockout model of MAO-B, in a paradigm of repeated open field exposure designed to assess habituation to novelty, reduced habituation was observed, suggesting that greater movement and novelty exploration are associated with reduced MAO-B level (Lee et al., 2004).

MAO-B density and activity are of considerable interest as indices of astrogliosis, and as abnormalities in several brain illnesses. Greater expression of MAO-B has been observed in astrocytes in many neuropsychiatric diseases such as Alzheimer’s disease and amyotrophic lateral sclerosis (Ekblom et al., 1993; Saura et al., 1994). This may account for the high number of neuropsychiatric diseases associated with elevated MAO-B density as well as therapeutics developed (see Table 1). However, in regard to therapeutics, it should be acknowledged that the MAO inhibitor discovery and development for MDD was originally serendipitous and based on observations that patients with tuberculosis receiving treatments with non-selective MAO inhibitor properties then had reductions in depressive symptoms. Interestingly, indices of the concentration of glial fibrillary acidic protein (GFAP), a sensitive and reliable marker of most, if not all, reactive astrocytes during brain disease, demonstrate high correlations with MAO-B binding in the primary regions assessed across postmortem investigations in Alzheimer’s disease, multisystem atrophy, progressive supranuclear palsy, and amyotrophic lateral sclerosis (Ekblom et al., 1993; Saura et al., 1994; Tong et al., 2017). Interestingly, assessments of MAO-B level in relation to other markers of astrocytosis such as vimentin and Hsp27 in the putamen of multisystem atrophy and progressive supranuclear palsy have also shown high correlations (Tong et al., 2017).

**TABLE 1:** Diseases with abnormal MAO-B levels/activity and therapeutics targeting MAO-B.

Disease	Abnormalities reported
Alzheimer’s disease	↑density, activity cortex, throughout brain to lesser extent (Saura et al., 1994)
Parkinson’s disease	↑density PFC, cell specific alterations in midbrain (Tong et al., 2017)
Progressive supranuclear palsy	↑density in caudate, frontal cortex, substantia nigra (Tong et al., 2017)
Multisystem atrophy	↑density in putamen (Tong et al., 2017)
Huntington’s disease	↑density, basal ganglia, pons, substantial nigra, insular cortex (Richards et al., 2011)
Amyotrophic lateral sclerosis	↑spinal cord (Ekblom et al., 1993)
Major depressive disorder	↑binding, prefrontal cortex (Moriguchi et al., 2019)
Alcohol use disorder	↑density PFC (Udemgba et al., 2014)
Cigarette smoking	↓binding globally in brain (Fowler et al., 1996a)
MPTP	Nigrostriatal tract
**Therapeutic**	**Intended diseases**
Phenelzine	Major depressive disorder
Tranylcypromine	Major depressive disorder
EMSAM (patch l-deprenyl)	Major depressive disorder
l-deprenyl	Parkinson’s disease
Rasagiline	Parkinson’s disease

## Development of Monoamine Oxidase B Imaging Agents

An important consideration is whether to develop a MAO-B PET imaging agent as an index of MAO-B density with irreversible or reversible binding or aim to develop a metabolic substrate of MAO-B to reflect an index of MAO-B activity. The density of MAO-B is highly correlated with its activity; with correlation coefficients typically > 0.9; plausibly due to minimal variation in two post-translational modifications to MAO-B (Saura et al., 1992, 1994, 1996; Tong et al., 2013). Hence measures of density are highly functionally relevant. Development of PET imaging agents for MAO-B has approached several of these directions.

**FIGURE 2:**
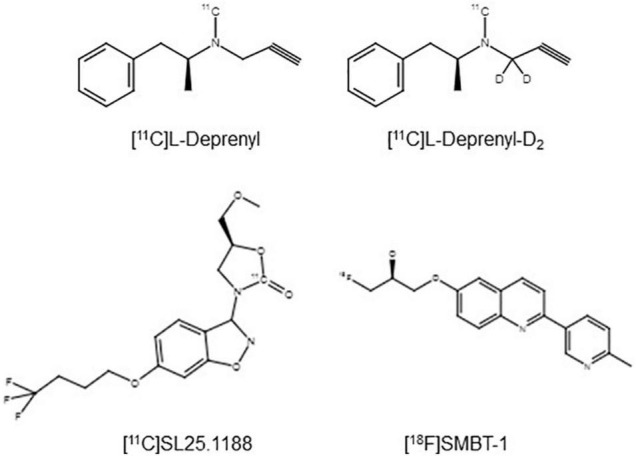
Chemical structures of PET imaging agents for MAO-B applied in humans (Fowler et al., 1995; Bramoulle et al., 2008; Harada et al., 2021).

Development of MAO-B PET imaging agents started with the strategy of applying radiotracers irreversibly bound to MAO-B so as to evaluate an index related to MAO-B density, as exemplified by [^11^C]L-deprenyl, [^11^C]L-deprenyl-D2, and [^18^F]fluorodeprenyl (also see Table 2). The advantage of this approach is that the density of the enzyme is largely related to the concentration of trapped radiotracer. A disadvantage is that excessive trapping results in irreversible time activity curves, which makes separating of parameters corresponding to radiotracer interactions with MAO-B from parameters corresponding to radiotracer delivery (and blood flow) difficult. The optimal window of radiotracer trapping vs. maintaining adequately reversible time activity curves to better quantify parameters of specific binding is difficult to achieve. [^11^C]L-deprenyl was the first PET MAO-B imaging agent applied in human brain *in vivo* (Fowler et al., 1987). Then to generate more reversible time activity curves, analogs were subsequently synthesized like deuterium labeled [^11^C]L-deprenyl ([^11^C]L-deprenyl-D2) (see Figure 2). [^18^F]deprenyl-D2 was also created and modeled in monkeys (Nag et al., 2016). The substitution of D2 reduced the rate of radiotracer binding by MAO-B leading to more reversible time activity curves. Another approach to obtain more information regarding specific binding parameters is to have a longer scanning period which was attempted with fluorination of deprenyl (with radiotracer [^18^F]fluorodeprenyl-D2; Nag et al., 2016). A limitation of this direction of radiotracer development is that [^11^C]L-deprenyl (as well as [^11^C]L-deprenyl-D2, etc.) are metabolized to brain penetrant radioactive metabolites which have additionally specific binding to dopamine and other monoamine transporters such as [^11^C]methamphetamine and [^11^C]amphetamine (Fowler et al., 1995). This complicates quantitation, especially in regions with high density of dopamine transporters like the striatum and substantia nigra.

**TABLE 2:** Comparison of PET imaging agents for central nervous system monoamine oxidase B applied in humans.

	[^11^C]L-deprenyl	[^11^C]L-deprenyl-D2	[^11^C]SL25.1188	[^18^F]-SMBT-1
**Affinity**	High (Nag et al., 2016) IC_50_ = ∼10 nM	Likely high* (Nag et al., 2016)	High (Bramoulle et al., 2008) K_i_ = 2 nM	High (Harada et al., 2021) K_D=_∼4 nM
**Selectivity**	High (Fowler et al., 1987)	High (Sturm et al., 2016)	High (Saba et al., 2010)	High (Harada et al., 2021)
**Reversibility**	Poor, includes one irreversible compartment (Fowler et al., 1995)	Low, includes one irreversible compartment (Fowler et al., 1995)	Excellent peak < 5 min (Rusjan et al., 2014)	Unknown in humans
**Brain uptake**	Excellent (Fowler et al., 1995) SUV∼4–5	Excellent (Fowler et al., 1995) SUV∼3–5	Excellent SUV∼5–6 (Rusjan et al., 2014)	Unknown in humans
**Modeling**	2 tissue compartments (Fowler et al., 1995); 1 reversible; 1 not	2 tissue compartments (Fowler et al., 1995); 1 reversible; 1 not	2 reversible tissue compartments (Rusjan et al., 2014)	Not published
**Specific binding** **	<0.2 very low (Fowler et al., 1995) λk’3E/λ; λ = V_*ND*_)	<0.1 very low (Fowler et al., 1995) (λk’3E/λ; λ = V_*ND*_)	7–12 very high (Rusjan et al., 2014) (V_*S*_/V_*ND*_)	Not known
**Reliability** (Mean absolute binding difference)	Good for whole brain; poor for regions (Jucaite et al., 2012)	<10% most regions <∼15% hippocampus over 1 day (Arakawa et al., 2017)	<15% over 6 weeks (Rusjan et al., 2014)	Not known
**Brain penetrant radioactive metabolites?**	[^11^C]methamphetamine, [^11^C]amphetamine (Fowler et al., 1995)	[^11^C]methamphetamine, [^11^C]amphetamine (Fowler et al., 1995)	Negligible (Rusjan et al., 2014)	Negligible (Harada et al., 2021)

*Deuterium labeling reduced the IC_50_ of [^18^F]fluorodeprenyl by an order of magnitude, it is plausible the effect would be similar for deuterium labeling of [^11^C]L-deprenyl (Nag et al., 2016).

**This ratio reflects the ratio of specific binding parameters calculated as compared to non-specific binding parameters calculated. For both reversible and irreversible imaging agents, this was V_ND_ reflecting the not specifically bound component (also referred to as λ in older source articles). Specific binding for reversible radioligands was V_S_. For the irreversible radiotracers, specific binding was k’3E.

**FIGURE 3:**
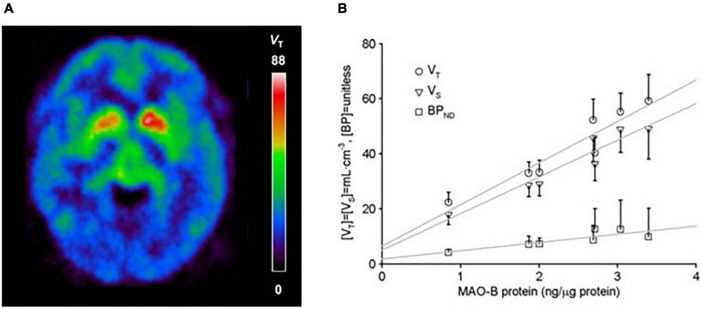
**(A)** [^11^C]SL25.1188 uptake in the human brain. Adapted with permission from Moriguchi et al. (2019). Copyright © (2019) American Medical Association. All rights reserved. **(B)** Relationship between regional average *in vivo* [^11^C]SL25.1188 *V_*T*_, V_*S*_*, and *BP*_*ND*_ (*n* = 14) and *in vitro* concentration of monoamine oxidase B (MAO-B) determined by immunoblotting in autopsied normal human brain (*n* = 6). Brain regions include cerebellar cortex, prefrontal cortex, temporal cortex, putamen, anterior cingulate cortex, thalamus, and caudate in order of increasing MAO-B protein concentrations. Dark lines represent SD. Linear fit analysis, *V_*T*_: r*^2^ = 0.9; *V_*S*_: r*^2^ = 0.92; *BP_*ND*_: r*^2^ = 0.7 (note *V*_*T*_ conventionally applied as index of MAO-B protein level). Adapted with permission of SAGE Publications from Rusjan et al. (2014). Copyright © (2014) ISCBFM.

A radioligand with an entirely different structure, [^11^C]SL25.1188 (see Figure 2), was discovered by Bramoulle et al. (2008) and subsequently modeled in baboons (Saba et al., 2010). This radioligand has highly reversible time activity curves giving considerable advantage for identifying parameters related to specific binding. The first production method for [^11^C]SL25.1188 required the toxic nerve gas phosgene (Bramoulle et al., 2008) making routine synthesis difficult. A new synthesis method involving CO_2_ fixation was discovered by Vasdev et al. (2011a,b), and then the radioligand was modeled in humans. [^11^C]SL25.1188 has outstanding properties for quantitative PET imaging, including high reversibility, brain uptake, and selectivity for MAO-B; no brain penetrant metabolites; and a very reproducible total V_T_ measure, an index of MAO-B level, which is highly correlated with the known concentration of MAO-B in postmortem human brain (*r*^2^ > 0.9) (see Figure 3 and Table 2; Rusjan et al., 2014).

A fluorinated analog of this compound, [^18^F] (S)-3-(6-(3-fluoropropoxy)benzo[d] isoxazol-3-yl)-5-(methoxymethyl)oxaz olidin-2-one, [^18^F] FSL25.1188 has also been synthesized, with high affinity, high brain uptake, and excellent reversibility in cynomolgus monkey and high selectivity in gray matter of the brain. Blocking studies suggested a lesser overall reduction of radioligand uptake in white matter leaving the possibility of either different free and non-specific binding in this tissue, or non-selectivity. Metabolites are polar, suggesting they are unlikely to be brain penetrant. However progressively increased radioactivity in bone after radioligand administration suggests that there is potential for bias from radioactivity in this structure for brain regions proximal to the skull. Overall, this radioligand has excellent potential for human study for brain regions not near to the skull (Dahl et al., 2019).

There is also continued interest in developing radiotracers that are metabolized such that the radioactive metabolite is trapped in the central nervous system. The advantage of this approach is that the function of the enzyme is largely related to the concentration of trapped radioactive metabolites. A disadvantage is that MAO-B is found throughout bodily organs, with the exception of the placenta, so the same metabolism occurs throughout the body, so the radioactive metabolites would likely be found in blood plasma as well as brain. This raises the question as to what extent the radioactive metabolites found in the brain are related to metabolism of the parent compound in the brain vs. radioactive metabolites in plasma crossing into the brain. There are exceptions to this concern, for example, if the radiotracer is trapped within the tissue and not distributed to plasma. This is exemplified with the radiotracer [^11^C]Cou for which the polar metabolite is trapped in tissue (Drake et al., 2018).

PET imaging agents metabolized by MAO-B with trapped radioactive metabolites is still an ongoing direction of development. This direction still shows intriguing promise given findings with the first radiotracer developed with PET, [^11^C]MPTP. [^11^C]MPTP had several useful properties, best demonstrated in its results in rhesus monkey showing high SUV; differentiation in uptake across regions consistent with regional variation in MAO-B density; and time activity curves that peaked reasonably early despite using a trapping mechanism to retain radioactive metabolites in the brain. However, this radiotracer was not intended for human use due to the severe and largely irreversible toxic effects associated with pharmacological doses of MPTP. The MPTP analog 4-methyl-7-((1-[^11^C]methyl-1,2,3,6-tetrahydropyridin-4-yl)oxy)-2H-chromen-2-one ([^11^C]Cou) was assessed in rhesus monkeys and found to be selectively metabolized by MAO-B; with the product of MAO-B oxidation and subsequent hydrolysis trapped in the brain (Drake et al., 2018). However, excessive trapping led to time activity curves that were insensitive to the relative density of MAO-B in the brain. Two deuterium substituted analogs, ([^11^C]Cou-d*3* and ([^11^C]Cou-d*7*) were created with the intention of reducing MAO-B metabolism further, by slowing down the hydrogen abstraction step, implicated as rate limiting in the MAO-B oxidation of MPTP, but the impact on time activity curves was fairly minimal, suggesting that these two analogs should not be developed further (Drake et al., 2018).

There is also continued interest in developing reversible MAO-B PET radioligands that are fluorinated to have longer half-lives so that the radioligands could be delivered to PET imaging centers without onsite cyclotrons. [^18^F]THK-5351 was designed to detect neurofibrillary tangles *in vivo* and was later found to bind MAO-B with high affinity (Villemagne et al., 2018). Unfortunately, the non-selective binding of [^18^F]THK-5351 to MAO-B and tau limits its utility for either purpose. However, a similar structured compound, (S)-(2-methylpyrid-5-yl)-6-[(3-18F-fluoro-2-hydroxy)propoxy]quinoline ([^18^F]-SMBT-1) (Figure 2) has been synthesized and applied in humans, although to date published validation studies are lacking (Harada et al., 2021; see Table 2). Selectivity of this latter compound for MAO-B was demonstrated in blocking studies in autoradiography of Alzheimer disease brains (which were positive for tau based on higher uptake of [^3^H]-MK6240; Harada et al., 2021).

It is noteworthy that there are evident problems for using reference tissue methods for MAO-B PET imaging agents. The distribution of MAO-B in the human brain is generally high ranging from 1 to 5 ng/μg protein (Tong et al., 2013). Relatively lower density regions include the cerebellum and white matter tissue, but MAO-B is still high in these tissues (Saura et al., 1996; Tong et al., 2013). Even for these regions, the MAO-B concentration is approximately half to a third of the concentration in the cortex and striatum, respectively, unfavorable ratios for use of a reference tissue (Saura et al., 1996; Tong et al., 2013). Accordingly, valid reference regions seem unlikely. If such regions were used as a reference like tissues for investigation into disease, it would also need to be assumed that there were no disease effects in the structure. Also, the MAO-B density is likely to contribute to a separate compartment in a reference tissue so it would seem plausible that reference tissue models accepting of more than one compartment in the reference tissue would likely be preferred as compared to single tissue compartment reference tissue approaches. Given these issues, clinical research presented largely excluded studies applying reference tissue methods.

## Clinical Research Applications

### Major Depressive Disorder

Prior to 2019, there were only two previous studies of MAO-B in the brain of MDD. These two studies measured [^3^H]lazabemide binding in overlapping sets of postmortem MDD cases: One sampled the dorsal raphe nucleus in 12 MDD subjects and the second sampled the amygdala in an extended sample of 15 MDD subjects (Klimek et al., 2003; Karolewicz et al., 2005). Both studies reported negative results, however, the sensitivity to detect an effect of diagnosis may have been reduced by concurrent cigarette smoking in 40% of the subjects, which lowers MAO-B binding (Fowler et al., 1996a). Inclusion of either early or late onset MDD may also have influenced findings since late onset MDD might include participants with neurodegenerative disease.

**FIGURE 4:**
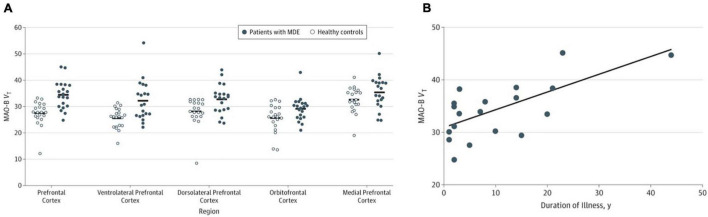
**(A)** Significantly elevated monoamine oxidase B total distribution volume (MAO-B V_*T*_) in the prefrontal cortex in patients during major depressive episodes (*n* = 20) compared with healthy controls (*n* = 20). **(B)** Relationship between prefrontal cortex MAO-B V_*T*_ and duration of illness in major depression (*n* = 20). Linear fit analysis, *r* = 0.68. Adapted with permission from Moriguchi et al. (2019). Copyright © (2019) American Medical Association. All rights reserved.

A case to study MAO-B in MDD was based on several arguments. During major depressive episodes (MDE), nuclear transcription factors associated with greater MAO-B transcription are elevated in the prefrontal cortex (Johnson et al., 2011; Harris et al., 2015). Also, glucocorticoids are often elevated during MDE, and glucocorticoid administration is associated with greater MAO-B expression and activity in the prefrontal cortex, as well as in cell lines (Slotkin et al., 1998; Lin et al., 2005; Chen et al., 2011; Kumar et al., 2011; Harris et al., 2015; Raitsin et al., 2017). Given these arguments, 20 participants with MDE without current psychiatric comorbidities and 20 age-matched healthy subjects underwent [^11^C]SL25.1188 PET scanning to evaluate MAO-B V_T_. All participants were drug and medication free, non-smoking, and otherwise healthy. Patients with MDE had a 26% greater MAO-B V_T_ in the prefrontal cortex, a highly significant finding (*P* = 0.0001) (Moriguchi et al., 2019; see Figure 4A). A key implication of this work is that the previously developed MAO inhibitor antidepressants which targeted MAO-A and MAO-B were viewed as having antidepressant properties due to their MAO-A inhibition. Given that elevation in MAO-B level itself occurs in the PFC during MDE, combined with the roles of MAO-B in generating oxidative stress, metabolism of neurotoxins, predisposition to astrogliosis, and metabolism of mood supporting monoamines, an important argument can be made that MAO-B inhibition is highly likely to be an important therapeutic property of the non-selective MAO inhibitor antidepressants (Moriguchi et al., 2019).

The second main outcome was to evaluate the relationship between MAO-B V_T_ and duration of illness (Moriguchi et al., 2019). A translocator protein binding PET study had demonstrated greater binding with greater duration of untreated MDD (Setiawan et al., 2018), a finding consistent with greater gliosis with greater duration of illness. Since astrogliosis is associated with MAO-B binding across several neuropsychiatric illnesses (Ekblom et al., 1994; Saura et al., 1994), it was anticipated that greater MAO-B binding would be greater in MDD with long duration of illness as was found across the 20 participants. More specifically, duration of MDD was positively correlated with MAO-B V_T_ in the prefrontal cortex and most subcortical regions including the thalamus (Moriguchi et al., 2019; see Figure 4B). Interestingly, the one cross-sectional study investigating the relationship of GFAP (a commonly applied marker of astrogliosis) with age and duration of illness in MDE found that the age-related increase in GFAP was much greater in MDD than healthy controls in the region sampled, the orbitofrontal cortex (Ekblom et al., 1993; Si et al., 2004). The association between greater MAO-B V_T_ and duration of illness is likely to reflect increasing astrogliosis in neuroprogressive MDD. Hence, future clinical trials targeting late-stage MDE should consider targeting MAO-B and astrogliosis as well as microglial activation.

### Cigarette Smoking

**FIGURE 5:**
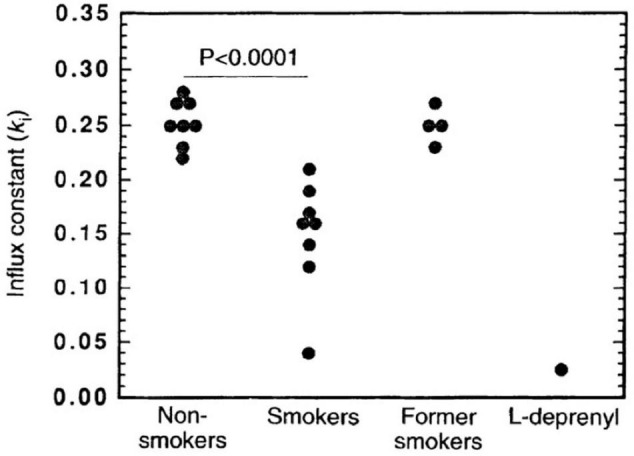
Significantly lower monoamine oxidase B (MAO-B) in smokers (*n* = 8) compared with non-smokers (*n* = 8) and former smokers (*n* = 4) as indicated by the effect on the influx rate constant (k_*i*_) for the basal ganglia. As a reference, data for one control that was given a therapeutic dose of the monoamine oxidase inhibitor, L-deprenyl (10 mgd^– 1^), for 1 week is included. Adapted with permission from Fowler et al. (1996a). Copyright © (1996) Nature Publishing Group.

A seminal study of Fowler et al. (1996a) demonstrated with [^11^C]L-deprenyl-D2 PET that MAO-B binding was lower by approximately 40% globally and throughout the brain regions assessed, including the basal ganglia, thalamus, cerebellum, cingulate gyrus, and frontal cortex (see Figure 5). The direction and magnitude of this finding is similar to the results of a later [^3^H]labamazide autoradiography study in postmortem tissue comparing cigarette smoking to non-cigarette smoking cases, in the amygdala (Karolewicz et al., 2005). Potential mechanisms to account for this difference include occupancy by a substance contained within cigarette smoke or an inherent underlying difference in the population of those who smoke cigarettes, although it is certainly possible other mechanisms influence the binding of [^11^C]L-deprenyl-D2 to MAO-B in the brain.

The favored mechanism by the investigators in this line of research to account for this difference is occupancy by a substance contained within cigarette smoke. However, there are other possibilities. For example, since the data was primarily derived from case control studies, an inherent underlying difference in the population of those who smoke cigarettes, such as a personality trait associated with low MAO-B density that also predisposes to smoking cigarettes could create some bias. This issue is well demonstrated in regards to the interpretation of MAO-A imaging studies in cigarette smoking given the large body of literature associating low MAO-A density and/or binding with impulsive behavior: Males with a rare point mutation in the eighth exon of the MAO-A gene that eliminates MAO-A demonstrate impulsive behavior (Brunner et al., 1993). Targeted knockout of MAO-A in mouse embryonic stem cells creates impulsively aggressive adult mice (Cases et al., 1995; Scott et al., 2008). Pharmacological inhibition of MAO-A during murine embryogenesis increases impulsive aggression in adult mice (Mejia et al., 2002). In addition, MAO-A genetic polymorphisms that are associated with lower MAO-A transcription in cell lines have been found to interact with childhood adversity to increase the risk of adult violent convictions (Caspi et al., 2002). Consistent with these findings, PET studies of healthy humans that used MAO-A-selective radioligands found inverse relationships between MAO-A binding in several brain regions, including the prefrontal cortex and self-reported impulsivity, aggression, and hostility (Alia-Klein et al., 2008; Soliman et al., 2011; Kolla et al., 2015). Initial studies of cigarette smoking reported that MAO-A binding is lower in cigarette smoking individuals, using a case control design which was interpreted as an effect of cigarette smoke. Since cigarette smoking individuals, on average, may have greater levels of impulsivity, some of this difference between cases and controls could be due to differences in the personality traits between groups. Even so, in longitudinal design it was shown that MAO-A availability changes temporally in a manner consistent with concordant change in MAO-A inhibiting beta carbolines and there was a high correlation shown between change in the beta carboline harmol with MAO-A availability (Fowler et al., 1996b; Bacher et al., 2011). In this example, personality trait of impulsivity may have created some bias leading to an overestimate of the effect attributable to cigarette smoking alone, but this bias did not completely account for the differences observed in the case control studies. There is an effect of cigarette smoking as well on MAO-A availability, but the magnitude attributable to cigarette smoke is likely lower than initial estimates.

To assess whether the difference in MAO-B binding in those who smoke cigarettes reflects a state effect of cigarette smoking vs. a trait difference in those who smoke cigarettes, a subsequent [^11^C]L-deprenyl D2 PET study applied a cross-sectional design to compare three groups. Participant groups were composed of 4 cases who had previously smoked cigarettes and then quit; 8 cases who currently smoke cigarettes and 8 cases who never smoked cigarettes. Across the regions assayed, the group who currently smoked cigarettes had lower MAO-B binding as compared to the other two groups which had similar MAO-B binding. Although it would have been better to have sampled a greater number of participants in each group, it was shown that the relationship between reduced MAO-B binding and cigarette smoking is state related (Fowler et al., 1998).

Applying [^11^C]L-deprenyl-D2 PET, the same research group also demonstrated that this finding of globally reduced MAO-B binding persists 12 h after self-reported cigarette cessation. However, the meaning of this finding is more difficult to interpret (Fowler et al., 2000a). There are a number of substances in cigarette smoke which inhibit MAO-B such as norharman, harmol, and 2-napthylamine (Herriaz and Chaparro, 2005) (it is interesting to note that the radioligand [^18^F]AV-1451 has a gamma carboline core, and some structural similarity to beta carbolines found in cigarette smoke (Drake et al., 2019)). However, such substances typically have low levels, and, given their half-lives, are undetectable after 12 h of withdrawal (Bacher et al., 2011). To account for the finding, it has been proposed that there may be an irreversibly binding substance in cigarette smoke that persistently interferes with MAO-B binding and/or catalytic activity. This explanation would concurrently explain reduced MAO-B expression in platelets after several weeks of cigarette smoking cessation (Fowler et al., 2000a). Another issue to consider in interpreting this study is compliance, monitored by plasma concentrations of several substances in cigarette smoke (Fowler et al., 2000a). As would be expected after cigarette cessation, plasma nicotine level was several-fold lower. However, neither expired carbon monoxide nor plasma levels of cotinine was lower. While the long half-life of cotinine at approximately 16 h might be less changed over 12 h, it is difficult to understand why CO level did not change, since its half-life is about 6 h, tends to correlate with changes in nicotine level; and would be expected to be lower after 12 h of cigarette cessation (Jarvik et al., 2000; Sandberg et al., 2011). It would be of interest to further study this complicated issue of MAO-B binding during cigarette withdrawal.

### Occupancy Studies

Neuroimaging with positron emission tomography (PET) to demonstrate target engagement through measuring occupancy hastens drug development. Occupancy may be defined as the percent change in specific binding after administration of a treatment intended to bind to the target. As one of the purported properties of gingko biloba is to inhibit MAO-B, based on assays of *in vitro* binding, its occupancy was assessed with [^11^C]L-deprenyl-D2 PET, after participants took ginkgo biloba extract EGb 761, 60 mg twice daily for a month. Changes in λk_3_, the index related to MAO-B binding of the radiotracer was unchanged, indicating no occupancy effects of ginkgo biloba *in vivo* (Fowler et al., 2000b). Dose-occupancy studies have also been conducted for experimental medications sembragiline and EVT301, demonstrating that high levels of occupancy are possible at doses which were well tolerated (Hirvonen et al., 2009; Sturm et al., 2016).

Rasagiline, a MAO-B inhibitor preferentially selective for MAO-B over MAO-A approved in many countries to treat Parkinson’s disease, was assessed for its occupancy and persistence of such after cessation of this medication. Three healthy volunteers received 1 mg of rasagiline daily for 10 days and then [^11^C]L-deprenyl PET was applied before dosing, immediately after dosing, and then after time periods of 2–3 and then 4–6 weeks (Freedman et al., 2005). The quantitation of radiotracer binding was conducted in a non-quantitative manner using a modified Patlak approach with a reference tissue. Although the measure was non-quantitative changes in time activity curves suggested brain penetration of rasagiline that persisted for 2–3 weeks beyond dosing. Interestingly, this is well beyond its removal from blood plasma but could be explained by either being an irreversible inhibitor and/or having a very long duration of clearance of either rasagiline or its main metabolite 1R-aminoindan from the central nervous system (Freedman et al., 2005). As rasagiline is an irreversible MAO-B inhibitor, some clarification regarding occupancy is warranted. The term “occupancy” used is defined as the percent change in the specific binding measure (quantitated with PET) after treatment. This definition was generally conceptualized for medications that bind reversibly to the target and was viewed as reflecting the proportion of sites blocked by the medication at the time of the second scan. In the case of an irreversibly binding drug, occupancy reflects a balance between removal of the specific binding sites by the irreversibly binding drug and ongoing protein synthesis to create new protein available to be specifically bound by the imaging agent. However, it is still a useful measure in the latter scenario as it still reflects available specific binding.

Overall, quantitative occupancy studies of MAO-B inhibitors are lacking for current MAO-B inhibitor therapeutics such as rasagiline, deprenyl, EMSAM, tranylcypromine, and phenelzine. Most occupancy investigations were supported by industry and occurred for therapeutics (or putative therapeutics) during their development such as EVT 301, sembragiline, and rasagiline in contrast to a number of medications already in use. The development of most medications in use, such as deprenyl, tranylcypromine, and phenelzine predate discovery of imaging methods to assess their occupancy. As a result, occupancies of established MAO-B inhibitor therapeutics are largely unknown. It is shown in other areas of PET occupancy therapeutics for central nervous system treatments that having standards can be useful, such as the establishment of 80% occupancy serotonin reuptake inhibitors (Meyer et al., 2004), which are then used as a standard for future serotonin reuptake inhibitors. Further work to understand the relative occupancy of currently used medications may assist in understanding the standards for successful therapeutics.

## Concluding Comments

MAO-B is an important brain protein given its high expression in the central nervous system; and its role in generating oxidative stress, facilitating monoamine metabolism, and influencing astrogliosis. Abnormalities of MAO-B density and/or activity are found in postmortem study of many diseases including Alzheimer’s disease (Saura et al., 1994), Parkinson’s disease (Tong et al., 2017), progressive supranuclear palsy (Tong et al., 2017), multisystem atrophy (Tong et al., 2017), Huntington’s disease (Richards et al., 2011), amyotrophic lateral sclerosis (Ekblom et al., 1993), and alcohol use disorder (Udemgba et al., 2014).

PET imaging developments for MAO-B largely followed strategies of irreversible and reversible binding compounds. [^11^C]L-deprenyl was the first radiotracer applied in human brain imaging of MAO-B with PET (Fowler et al., 1987), but to reduce the impact of poor reversibility, [^11^C]L-deprenyl-D2 was created. [^11^C]L-deprenyl-D2 has limitations of poor reversibility, and radioactive metabolites with specific binding for dopamine and norepinephrine transporters. It is particularly difficult to make imaging agents with a trapping compartment for the parent compound or metabolites and, at the same time, both maintain reversible time activity curves in brain tissue and avoid similar metabolites being generated in the periphery, as MAO-B is found through the body. However, this does not preclude the possibility of accomplishing this, and efforts continue with radiotracers such as analogs of [^11^C]Cou. In contrast, [^11^C]SL25.1188 is an outstanding radioligand with a specific binding to non-displacement ratio of 7–12, high selectivity, high reversibility, and no evidence for substantial brain penetrant metabolites. Its main disadvantage is that just like [^11^C]L-deprenyl-D2, quantitation presently requires arterial blood sampling.

Important clinical research applications have been completed with MAO-B imaging. The elevation in MAO-B V_*T*_ of over 1.5 standard deviations in the prefrontal cortex during MDE of MDD is quantitatively substantive, and, in combination with the roles for MAO-B in generating oxidative stress, metabolizing monoamines, and apoptosis, makes a case that the MAO-B inhibition of MAO inhibitors, not just the MAO-A inhibition has important therapeutic implications. Regardless of the limitations of [^11^C]L-deprenyl-D2, the interpretation of a lesser level of MAO-B binding in those who smoke cigarettes is solid and replicable. The mechanism underlying this interesting finding is uncertain although irreversible binding by a metabolite of cigarette smoke is a plausible mechanism. It was demonstrated that gingko biloba does not occupy MAO-B, and that sembragiline occupies a high proportion of MAO-B sites, whereas the occupancy study of rasagaline did not use a validated method of quantitation. Hence the main implication of this latter study is that rasagiline shows brain penetration of drug that persists over a longer time course than its removal from plasma, with the numerical level of occupancy being uncertain. While several important clinical applications have been completed with MAO-B imaging, given the number of neuropsychiatric diseases with abnormal MAO-B density and/or activity, and the role of MAO-B in astrogliosis, it is evident there are opportunities for more discovery and drug development.

## Author Contributions

JM: conception and primary draft manuscript preparation. JB: draft manuscript preparation. Both authors contributed to the article and approved the submitted version.

## Conflict of Interest

JM has received operating grant funding from the Government of Ontario with Janssen for studies of translocator protein binding in relation to blood biomarkers. JM also has patents for blood biomarkers in mood disorders to predict neuroinflammation and elevated MAO-B in the brain and a patent for a dietary supplement to prevent post-partum depression. JM was also developing MAO-B inhibitors for use in neuropsychiatry. The remaining author declares that the research was conducted in the absence of any commercial or financial relationships that could be construed as a potential conflict of interest.

## Publisher’s Note

All claims expressed in this article are solely those of the authors and do not necessarily represent those of their affiliated organizations, or those of the publisher, the editors and the reviewers. Any product that may be evaluated in this article, or claim that may be made by its manufacturer, is not guaranteed or endorsed by the publisher.
